# Treatment, long-term outcome and prognostic variables in 214 unselected AML patients in Sweden

**DOI:** 10.1054/bjoc.1999.1123

**Published:** 2000-03-21

**Authors:** M Åström, L Bodin, I Nilsson, U Tidefelt

**Affiliations:** Division of Haematology, Department of Internal Medicine, Örebro Medical Center Hospital, Örebro, S-701 85, Sweden; Unit of Biostatistics and Epidemiology, Örebro Medical Center Hospital, Örebro, S-701 85, Sweden; Department of Internal Medicine, Karlstad Central Hospital, Karlstad, S-651 85, Sweden

**Keywords:** AML, survival, selection, prognostic factors

## Abstract

With the aim of describing an unselected series of acute myeloid leukaemias (AML) in adults, patients diagnosed 1987–1992 in the Örebro region of central Sweden were reviewed by investigating hospital records. By utilizing: (1) The Swedish Cancer Registry, (2) The Cause of Death Registry, (3) listings of pathology bone marrow reports and (4) listings of inpatient discharge diagnoses, we attempted to find all patients. Among secondary AML, only blast-crises of CML were excluded. A total of 214 cases of AML with a median age of 69.5 years were verified corresponding to a mean yearly incidence in adults of 5.4/100 000. Of all patients, 56% had received ‘high-dose’ induction treatment, 28% ‘low-dose’ treatment and 16% no cytostatic treatment. Median survival for all patients was 5.8 months and the probability of survival at 5 years was 9.3%. The 120 ‘high-dose’ treated patients had a total CR rate of 67%, median CR duration 10.1 months and median survival 11.4 months. Age, LDH and kidney function were found to be independent prognostic variables for survival. The inclusion of patients unreferred from district hospitals makes this study unique as an example of unselected AML. © 2000 Cancer Research Campaign


British Journal of Cancer (2000) **82**(8), 1387–1392
© 2000 Cancer Research Campaign
DOI: 10.1054/ bjoc.1999.1123, available online at http://www.idealibrary.com on IDEAL®

### Treatment, long-term outcome and prognostic variables in 214 unselected AML patients in Sweden

**M Åström[1], L Bodin[2], I Nilsson[3] and U Tidefelt[1]**

[1]Division of Haematology, Department of Internal Medicine, Örebro Medical Center Hospital, S-701 85 Örebro, Sweden; [2]Unit of Biostatistics and Epidemiology, Örebro Medical Center Hospital, S-701 85 Örebro, Sweden; [3]Department of Internal Medicine, Karlstad Central Hospital, S-651 85 Karlstad, Sweden

**Summary** With the aim of describing an unselected series of acute myeloid leukaemias (AML) in adults, patients diagnosed 1987–1992 in the Örebro region of central Sweden were reviewed by investigating hospital records. By utilizing: (1) The Swedish Cancer Registry, (2) The Cause of Death Registry, (3) listings of pathology bone marrow reports and (4) listings of inpatient discharge diagnoses, we attempted to find all patients. Among secondary AML, only blast-crises of CML were excluded. A total of 214 cases of AML with a median age of 69.5 years were verified corresponding to a mean yearly incidence in adults of 5.4/100 000. Of all patients, 56% had received 'high-dose' induction treatment, 28% 'low-dose' treatment and 16% no cytostatic treatment. Median survival for all patients was 5.8 months and the probability of survival at 5 years was 9.3%. The 120 'high-dose' treated patients had a total CR rate of 67%, median CR duration 10.1 months and median survival 11.4 months. Age, LDH and kidney function were found to be independent prognostic variables for survival. The inclusion of patients unreferred from district hospitals makes this study unique as an example of unselected AML. © 2000 Cancer Research Campaign

**Keywords**: AML; survival; selection; prognostic factors

Overall prognosis in acute myeloid leukaemia (AML) is difficult to predict from previous reports, since these are usually dealing with selected cohorts of patients (Boros et al, 1985; The Toronto Leukemia Study Group, 1986; Wahlin et al, 1991; Ferrara et al, 1998). Elderly patients are often excluded from treatment trials for various reasons (The Toronto Leukemia Study Group, 1986; Ferrara et al, 1998). Differences in referral patterns make overall treatment results from specialized centres difficult to interpret (Taylor et al, 1995). We know few studies attempting to give the outcome of treatments on the basis of an epidemiologic definition of AML. Even in these studies there is probably some selection of patients, since they were based on cases treated at specialized centres or reported to an official registry (Öst et al, 1984; Brincker, 1985; Wahlin et al, 1991; Proctor et al, 1995; Taylor et al, 1995). It has been shown that official statistics of morbidity and mortality is not always reliable concerning the total occurrence of haematological malignancies (Mattsson and Wallgren, 1984).

We decided to do a retrospective review of the treatment and outcome of unselected adult AML patients in our geographical region, including patients unreferred from district hospitals. By using four independent registries, the coverage of patients has been maximized. We also attempted to assess the prognostic value of factors such as age, treatment, antecedent haematological disorder and laboratory characteristics in this material.

### MATERIALS AND METHODS

### Population

The population base for this investigation consists of three counties of similar size in central Sweden: Örebro, Södermanland and Värmland. During the study period, 1987–1992, the total number of inhabitants in this region rose from 797 960 to 816 874, of whom 657 397 and 667 496 respectively were 15 years or older. Children under 15 years were not included in the investigation. The age distribution was somewhat skewed compared to the national average, with 24.5% over 60 years in our region compared with 22.8% in December 1989. Exact demographic data are available for each year of the study period (Statistics Sweden, 1987–1992). All patients with AML who were domiciled in any of the three counties of the study were included, even those few who received treatment at specialized clinics outside the geographical region.

*Received 16 June 1999*
*Revised 10 November 1999*
*Accepted 15 November 1999*

*Correspondence to:* M Åström

### Patients

Our method for case-finding consisted of a comparison of four different registries: (1) The Swedish Cancer Registry, (2) The Cause of Death Registry, (3) listings of pathology bone marrow reports, and (4) listings of inpatient discharge diagnoses. All included cases were adults found with newly diagnosed AML 1987–1992. Among secondary AML, only blast-crises of chronic myeloid leukaemia (CML) were excluded, whereas leukaemias secondary to other haematological conditions, chemotherapy or irradiation were included. A total of 214 cases of AML were verified from medical records, corresponding to a mean yearly incidence in adults of 5.4/100 000. The median age of the patients was 69.5 years. The age distribution is shown in Figure 1, with 74% over 60 years at diagnosis, 52% over 70 years, 5% under 30 years.

### Statistical methods

Study results were last updated on 31 December 1997. For the evaluation of factors of importance for complete remission (CR) frequency, contingency tables describing the CR frequency stratified with respect to the investigated factors were analysed with Fisher's exact test. Prognostic factors for overall survival and CR

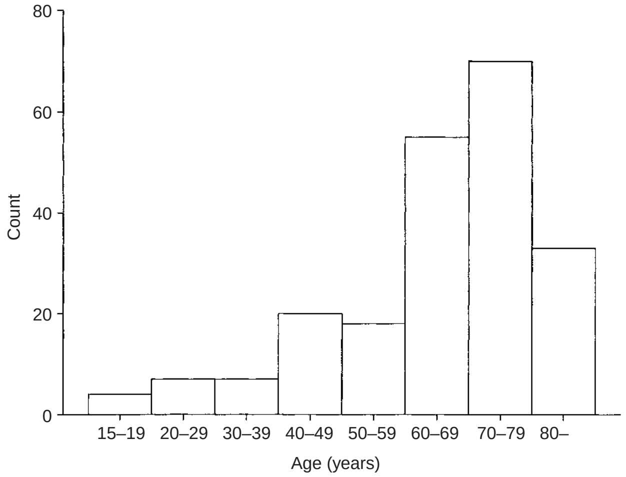

**Figure 1** Age distribution of 214 unselected AML cases from the Örebro region of central Sweden diagnosed 1987–1992

duration were found by calculating Kaplan–Meier curves, comparing subgroups with the log-rank test. Prognostic variables from univariate analyses were also entered into multivariate analyses using Cox proportional hazard regression with a forward stepwise selection procedure. Reduced kidney function was defined as creatinine values above the upper reference limits in our laboratory for males and females respectively. A *P*-value ≤ 0.05 was regarded as statistically significant in all analyses.

### RESULTS

### FAB classification and cytogenetics

The AML diagnoses were based upon stained bone marrow smears in 210 cases where four were taken at autopsy, and peripheral blood in four cases. Information about French–American–British (FAB) class (Bennett et al, 1985) was found in clinical records and bone marrow reports in 155 of the 214 AML cases: M0 *n* = 3, M1 *n* = 40, M2 *n* = 47, M3 *n* = 9, M4 *n* = 33, M5 *n* = 19, M6 *n* = 3, M7 *n* = 1. Unclassified patients had a median age of 74.9 years and the proportion of secondary AML was 41% among these cases. Diagnostic confirmation by immunophenotyping and/or cytochemistry had been performed in 73% of 'high-dose'-treated patients, compared to 40% of 'low-dose'-treated patients and 9% of untreated patients. Cytogenetic examinations had been successfully performed in 49 cases of whom 38 were 'high-dose'-treated. A normal karyotype was found in 28 cases and 21 had clonal abnormalities. Only three cases had translocations t(8;21) or t(15;17) which are related to good prognosis in AML, but in these other abnormalities were present concomitantly. Of patients under 60 years, 7/24 had chromosomal abnormalities compared to 14/25 of patients 60 years or older, with more complex abnormalities in the oldest patients.

### Secondary AML

Secondary AML was present in 48 cases with the median age 72.7 years, of whom 28 had histories of myelodysplastic syndromes (MDS). Myeloproliferative disease, lymphoma or myeloma had been present in 16 patients and four patients with antecedent solid cancer forms had received irradiation treatment. In addition, 19 patients of whom 13 were over 60 years old had trilinear dysplasia documented in bone marrow at diagnosis, without a history of MDS.

### Cytostatic treatment

One hundred and twenty patients with a median age of 61.1 years had received 'high-dose' induction regimens aiming at obtaining complete remissions, 60 'low-dose' treatments and 34 only palliative care. Decisions to refrain from intensive chemotherapy in many elderly patients were made by individual doctors and their patients. For 'high-dose'-treated patients, induction therapy consisted of ara-C in standard or intermediate doses (79 cases) or doses over 1 g $m^{-2}$ (41 cases) in combination with an anthracycline or mitoxantrone (118 cases). In 107 courses also thioguanine or etoposide was included.

Of 83 patients achieving CR, at least one consolidation course was given to 43 patients. Four of these patients were also treated with allogenic bone marrow transplantation (BMT) and seven with autologous bone marrow transplantation (ABMT) in first CR. The remaining 40 patients, mostly elderly, received 'low-dose' maintenance treatment with thioguanine (20–320 mg $week^{-1}$ orally given on 1–2 consecutive days) continued until relapse or for at least 3 years in relapse-free patients. Of 61 relapses in the total material, 48 were treated with new 'high-dose' induction courses, in two cases followed by BMT and in three cases by ABMT.

Of the 60 'low-dose'-treated patients, seven had received reduced induction courses in order to avoid toxicity. The remainder of the 'low-dose'-treated patients had been managed

**Table 1** Treatment types in different age categories and corresponding CR frequencies, CR durations and survival

| | All | 'High-dose' treatment | | | | 'Low-dose' treatment | | | | No treatment | |
|---|---|---|---|---|---|---|---|---|---|---|---|
| Age | *n* | % | CR frequency % | CR duration[a] | Survival[a] | % | CR frequency % | CR duration[a] | Survival[a] | % | Survival[a] |
| –59 | 56 | 98 | 80 | 13.7 | 24.9 | 2 | 0 | – | 8.3 | – | – |
| 60–69 | 55 | 78 | 84 | 7.0 | 10.4 | 13 | 14 | 0.9 | 4.6 | 9 | 0.2 |
| 70–79 | 70 | 29 | 60 | 6.1 | 8.6 | 50 | 3 | 2.3 | 2.7 | 21 | 0.2 |
| 80– | 33 | 6 | 50 | 10.0 | 10.9[b] | 52 | 6 | 7.9 | 2.4 | 42 | 0.4 |
| All ages | 214 | 56 | 67 | 10.1 | 11.4 | 28 | 5 | 2.3 | 2.9 | 16 | 0.3 |

[a]Median values in months. [b]Only two patients with survival times 0.3 and 21.5 months respectively.

© 2000 Cancer Research Campaign

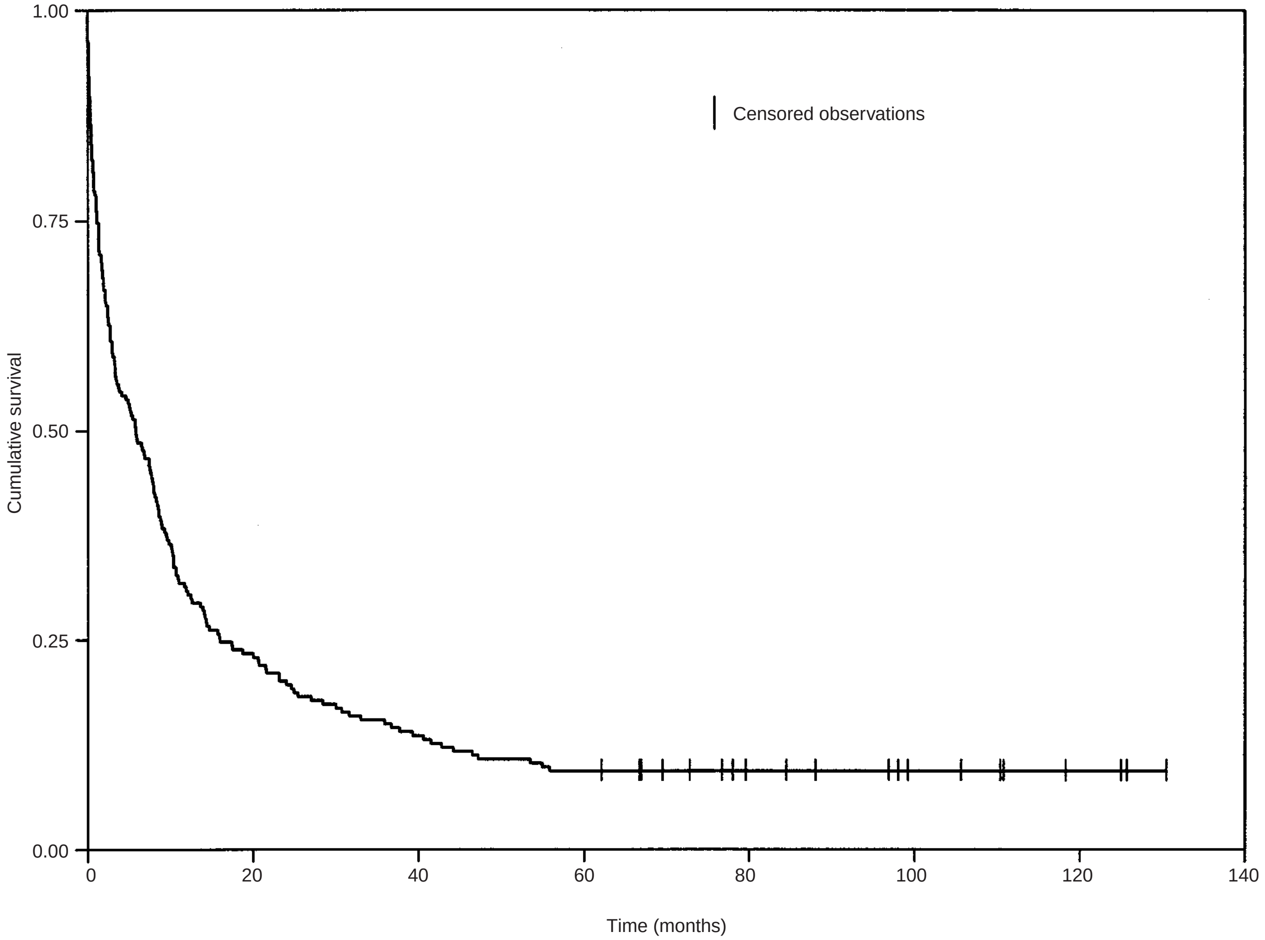

**Figure 2** Overall survival for 214 unselected patients with AML in the Örebro region of central Sweden 1987–1992. Median age is 69.5 years, median survival is 5.8 months, probability of survival at 5 years is 9.3%

with palliative intention mainly with ara-C, thioguanine and etoposide separately or in combination.

### Outcome

Overall survival in all 214 patients without exclusions is shown in Figure 2. Median survival was 5.8 months and the probability of survival at 5 years was 9.3%. CR rate for all patients was 38.8% and the median duration of the first remission was 9.8 months. In the patients acheiving CR, median survival was 21.6 months compared to 1.8 months in those who did not achieve CR.

When separated according to type of treatment and age it is evident that patients receiving 'high-dose' induction chemotherapy had the best outcome (Table 1). The 120 'high-dose'-treated patients had a CR rate of 67%, median CR duration 10.1 months and median survival 11.4 months. Only one patient under 60 years of age received 'low-dose' treatment for MDS-AML. In contrast, a majority of the patients over 70 years were given 'low-dose' or no cytostatic treatment. For all the 60 'low-dose'-treated patients, median survival was 2.9 months compared to 0.3 months for patients receiving no chemotherapy at all.

### Age and prognosis

Survival according to age irrespective of treatment is illustrated in Figure 3. The median survival time for 56 patients under 60 years was 24.8 months compared to 3.1 months for 158 patients 60 years or older. Survival times also differed significantly between ages 60–69, 70–79, and over 80 years. There were 55, 70 and 33 patients in these age groups with median survival times 8.4, 2.4 and 1 month respectively. Survival at 5 years was 45% in the youngest age group under 30 years, 24% in patients 30–59 years, 5% in patients 60–69 years, 1% in those 70–79 years and 0% above 80 years. The poorer survival in the elderly correlated with both lower CR rates and shorter CR duration. Among patients under 60 years the CR rate was 79% and CR duration 13.7 months. In patients over 60 years the CR rate was 24% and CR duration 6.8 months.

### Other prognostic variables

When specifically assessing the influence of variables on CR frequency, CR duration and survival, only 'high-dose'-treated patients were included in the evaluation. There were no significant differences in outcome depending on FAB class, whereas patients

*© 2000 Cancer Research Campaign*

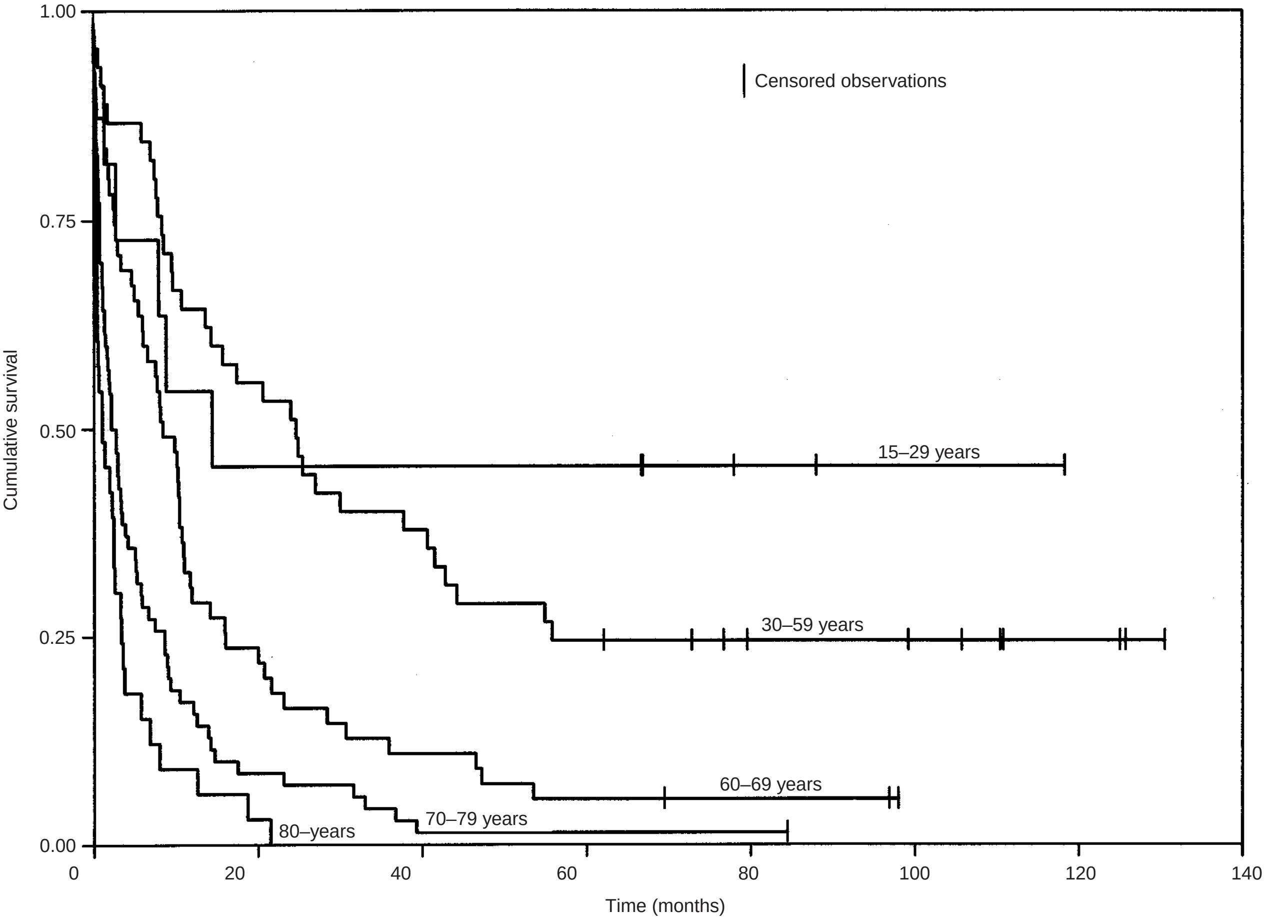

**Figure 3** Overall survival in 214 unselected AML patients depending on age. The median survival was 24.8 months for patients under 60 years compared to 3.1 months in those 60 years or older. The difference in survival is significant ($P < 0.0001$)

with chromosomal abnormalities had lower CR frequency ($P = 0.02$) and survival ($P = 0.02$). Other factors with a negative impact on CR frequency were age over 60 years ($P = 0.01$), presence of secondary AML ($P = 0.05$) and impaired kidney function at diagnosis ($P = 0.05$). Overall survival was poorer in patients over 60 years ($P = 0.0002$), with secondary AML ($P = 0.04$), impaired kidney function ($P = 0.05$) or lactate dehydrogenase (LDH) values above 25 μkat $l^{-1}$ ($P = 0.05$). A leucocyte count above $100 \times 10^9 l^{-1}$ was statistically non-significant for shorter survival ($P = 0.07$). A multivariate analysis was performed concerning prognostic variables for survival, where results of cytogenetics were excluded from the analysis due to many missing cases. The included variables were age, LDH, leucocyte count, kidney function and presence of de novo/secondary leukaemia. The only prognostic variables found in the stepwise selection procedure were age ($P = 0.005$), LDH ($P = 0.02$) and kidney function ($P = 0.05$).

Concerning CR duration, the only significant variable in both univariate and multivariate analysis was treatment intensity in CR where the 43 patients who received consolidation therapy fared better than the 40 who were given 'low-dose' maintenance treatment only ($P < 0.0001$). This was evident also in the higher age group over 60 years. Median CR duration for all patients treated with consolidation was 24.7 months compared to 5.4 months for those who received 'low-dose' treatment in first CR.

### Selection in specialized clinics

Survival of 149 patients treated at Örebro Medical Center Hospital or in nine cases other specialized clinics was compared to survival of 37 patients in two central hospitals and 28 patients in eight district hospitals. The median age of the 149 patients treated in specialized clinics was 66.0 years, compared to 75.4 years in central hospitals and 79.3 years in district hospitals. The corresponding median survival times were 8.3 months, 2.9 months and 1.5 months respectively. Of the 65 patients treated at non-specialized centres, only 12 received 'high-dose' induction treatments, all at the Central Hospital in Karlstad, with no difference in survival and treatment-related mortality compared to similar risk patients treated in Örebro.

### Early deaths

The frequency of 'early death', within 1 month from diagnosis in 'high-dose'-treated patients was 10/120 or 8.3%. In four cases the cause was intracerebral haemorrhages, all these patients having

 *© 2000 Cancer Research Campaign*

disseminated intravascular coagulation at diagnosis. Another patient died of a cerebral thrombosis, possibly on the basis of leucostasis at presentation. In two cases, myocardial infarction in connection with induction therapy was the terminal cause of death. One patient died on the second day of admission with respiratory failure from rapidly proliferative leukaemia. Additionally, two patients died of infection and of multiorgan failure respectively.

### DISCUSSION

Unlike most other studies of AML which are based on patients treated at specialized hospitals, the present investigation is a retrospective review covering also patients unreferred to such centres. Our aim was to describe the full clinical spectrum of AML as well as referral patterns, treatment decisions and outcome. In another part of our study we have documented an undernotification of acute leukaemias in the Swedish Cancer Registry of 15.4% among adults and inconsistent coding in an additional 8.1% for the same period 1987–1992. Overall, we wanted to make a quality assessment of the management of AML in our geographical region, as an example of a method of evaluation.

In comparison with earlier studies of AML, our incidence figure of 5.4/100 000 per year in adults is high. If the two patients under 15 years with AML from our region in the Cancer Registry 1987–1992 would be included in the calculation, the yearly incidence for all ages would amount to 4.5/100 000. Official crude incidence rates for AML from the Swedish Cancer Registry 1987–1992 were consistently in the range 3.1–3.4/100 000 per year for the total Swedish population, with corresponding ‘world standardized rates’ (WSR) of around 2.0/100 000. Studies from other European countries often show similar incidence rates as those from the Swedish Cancer Registry, for example a study from England and Wales with 3.4/100 000 per year (McKinney et al, 1989). We believe that the higher incidence figure in our study, achieved by utilizing multiple sources of notification, illustrates a common problem of undernotification in Cancer Registries and selection in clinical trials.

That AML is a disease predominantly of the elderly is known from earlier studies, with more than half of the cases being diagnosed in patients over 60 years (Öst et al, 1984; Brincker, 1985; McKinney et al, 1989; Wahlin et al, 1991). In the present investigation, 74% of the patients were over 60 years. A rising incidence of AML depending on the increasing longevity of the population is to be expected in the future, necessitating a reconsideration of treatment priorities and resource allocation where attention must be given to solving the problems of treatment in elderly patients (Taylor et al, 1995; Hoff et al, 1997).

Immunophenotyping and cytogenetics had been utilized in approximately the same frequency at Örebro Medical Center Hospital as in other studies from specialized centres in the near time period (Wahlin et al, 1991; Bassan et al, 1992; Proctor et al, 1995; Taylor et al, 1995). A majority of all bone marrow specimens were analysed in Örebro Medical Center Hospital by either haematologists or haematopathologists, but in the period 1987–1989 specimens were also sent to another centre for cytochemistry. From patients unreferred to our hospital, smaller pathology departments at the two other central hospitals had the responsibility for diagnostics on bone marrow material. In the total material, FAB classification was not always attempted and especially in many MDS-AML cases not deemed to be of interest.

According to recent literature on AML, approximately 60–80% of patients less than 60 years of age can be expected to achieve CR (Rowe and Liesveld, 1996). For patients over 60 years, studies report varying CR frequencies from 40 to 70% using comparable induction regimens and essentially identical supportive treatment (Löwenberg, 1996). Preselection of patients may have led to over-optimistic results in some of these trials. In studies in which consecutive patients have been evaluated, a less favourable but more realistic reality exists in terms of CR attainment and survival (The Toronto Leukemia Study Group, 1986; Bassan et al, 1992; Baudard et al, 1994).

In our material of unselected AML, 98% of patients under 60 years received intensive chemotherapy, compared to 78% of patients aged 60–69 years, 29% of patients 70–79 years and 6% of patients over 80 years. The relatively high CR rates 80%, 84%, 60% and 50% in the treated patients thus correlate to a marked selection in the higher age groups. Secondary AML patients received ‘low-dose’ treatment more often than those with de novo AML. How factors such as poor performance status and concomitant disease have influenced decisions to refrain from intensive chemotherapy cannot be quantified in this material. We do not know if the high exclusion rate from induction chemotherapy had a good or bad impact on our total survival. Median overall survival was higher than in previous population-based reports from Sweden with 24.8 months for patients under 60 years and 3.1 months for those over 60 years, including unreferred and untreated patients (Öst et al, 1984; Wahlin et al, 1991).

For patients over 80 years, who in our study constituted 15% of all AML cases, an adverse effect of intensive chemotherapy on survival and overall quality of life has been indicated in an earlier study from the MD Anderson Cancer Center (DeLima et al, 1996). In our experience, low-dose chemotherapy with thioguanine, ara-C and etoposide alone or in combination without intention to induce cytopenia has been of value in some patients, at least in alleviating symptoms of a high leukaemic cell burden. Whether or not such therapy prolongs survival significantly in larger patient materials is under question as AML treatments with palliative intention have not been studied in a randomized way. Other investigational therapies would also be of interest in this age group (DeLima et al, 1996).

In spite of the heterogeneity of our patient material, we could confirm the value of some established prognostic factors in AML in our intensively treated patients. Age has proved to be the most important factor in many studies (Bernard et al, 1984; Estey et al, 1989; Wahlin et al, 1991). The prognostic impact of age per se is difficult to evaluate in this study as treatment decisions were largely based on age and performance status of the patients, without uniform documentation of the latter. Karyotype has emerged as the most important feature of AML concerning prognosis, and already has therapeutic consequences in modern AML treatment (Bernard et al, 1984; Büchner and Heinecke, 1996; Fenaux et al, 1989; Ferrara et al, 1998). Secondary AML had worse outcome than de novo AML but did not appear as a selected prognostic factor in the present investigation probably due to a strong correlation to the patient age.

Among routinely measured laboratory variables, leucocyte count (Dutcher et al, 1987; Fenaux et al, 1989), LDH (Büchner and Heinecke, 1996; Ferrara and Mirto, 1996) and kidney function tests (Estey et al, 1989; Johnson et al, 1993, 1995) have repeatedly been assigned prognostic value. Of such variables, LDH and creatinine appeared somewhat stronger than the leucocyte count in the

© 2000 Cancer Research Campaign present study, reaching independent significance for survival. No correlation was seen between these variables and treatment intensity. Nearly 1/4 of all patients had impaired kidney function at diagnosis, often unexplained by medical history and reversible with chemotherapy, suggesting a contributing role of the leukaemia itself. The prognostic value of kidney function tests in AML is probably not only related to concomitant other disease or poor performance status.

In summary, we believe our study to be a unique example of unselected AML cases. Although the long-term survival at 5 years was as low as 9.3%, some improvement in survival is shown in comparison with similar earlier studies. The treatment of an increasing proportion of elderly patients will be a challenge in the future, where the crucial question of how to predict benefit or not from intensive chemotherapy needs to be further investigated.

### ACKNOWLEDGEMENTS

This study was supported by grants from the Örebro County Research Committee and the Swedish Cancer Society. We thank doctors and administrative staff at all involved hospitals for their kind and enduring cooperation.

### REFERENCES

Bassan R, Buelli M, Viero P, Minotti C and Barbui T (1992) The management of acute myelogenous leukemia in the elderly: ten year experience in 118 patients. *Hematol Oncol* **10**: 251–260

Baudard M, Marie JP, Cadiou M, Viguié F and Zittoun R (1994) Acute myelogenous leukaemia in the elderly: retrospective study of 235 consecutive patients. *Br J Haematol* **86**: 82–91

Bennett JM, Catovsky D, Daniel MT, Flandrin G, Galton DAG, Gralnick HR and Sultan C (1985) Proposed revised criteria for the classification of acute myeloid leukemia. A report of the French–American–British Cooperative Group. *Ann Int Med* **103**: 620–625

Bernard P, Reiffers J, Lacombe F, Dachary D, Boisseau M and Broustet A (1984) A stage classification for prognosis in adult acute myelogenous leukemia based upon patients' age, bone marrow karyotype and clinical features. *Scand J Haematol* **32**: 429–440

Boros L, Chuang C, Butler FO and Bennett JM (1985) Leukemia in Rochester (NY). A 17-year experience with an analysis of the role of cooperative group (ECOG) participation. *Cancer* **56**: 2161–2169

Brincker H (1985) Estimate of overall treatment results in acute non-lymphocytic leukemia based on age-specific rates of incidence and of complete remission. *Cancer Treat Rep* **69**: 5–11

Büchner T and Heinecke A (1996) The role of prognostic factors in acute myeloid leukemia. *Leukemia* **10**: 28–29

DeLima M, Ghaddar H, Pierce S and Estey E (1996) Treatment of newly-diagnosed acute myelogenous leukaemia in patients aged 80 years and above. *Br J Haematol* **93**: 89–95

Dutcher JP, Schiffer CA and Wiernik PH (1987) Hyperleukocytosis in adult acute nonlymphocytic leukemia: impact on remission rate and duration, and survival. *J Clin Oncol* **5**: 1364–1372

Estey E, Smith TL, Keating MJ, McCredie KB, Gehan EA and Freireich EJ (1989) Prediction of survival during induction therapy in patients with newly diagnosed acute myeloblastic leukemia. *Leukemia* **3**: 257–263

Fenaux P, Preudhomme C, Lai JL, Morel P, Beuscart R and Bauters F (1989) Cytogenetics and their prognostic value in de novo acute myeloid leukemia: a report on 283 cases. *Br J Haematol* **73**: 61–67

Ferrara F and Mirto S (1996) Serum LDH value as a predictor of clinical outcome in acute myelogenous leukaemia of the elderly. *Br J Haematol* **92**: 627–631

Ferrara F, Mirto S, Zagonel V and Pinto A (1998) Acute myeloid leukemia in the elderly: a critical review of therapeutic approaches and appraisal of results of therapy. *Leuk Lymphoma* **29**: 375–382

Hoff PM, Pierce S and Estey E (1997) Comparison of acute myeloid leukemia patients at MD Anderson: 1982–1986 vs 1992–1996. *Leukemia* **11**: 1997–1998

Johnson PRE, Hunt LP and Liu Yin JA (1993) Prognostic factors in elderly patients with acute myeloid leukaemia: development of a model to predict survival. *Br J Haematol* **85**: 300–306

Johnson PRE, Ryder WD and Liu Yin JA (1995) Validation of a model to predict survival in elderly patients with acute myeloid leukaemia. *Br J Haematol* **90**: 954–956

Löwenberg B (1996) Treatment of the elderly patient with acute myeloid leukaemia. *Baillières Clin Haematol* **9**: 147–159

Mattsson B and Wallgren A (1984) Completeness of the Swedish Cancer Register. *Acta Radiol Oncol* **23**: 305–313

McKinney PA, Alexander F, Cartwright RA and Ricketts TJ (1989) The Leukaemia Research Fund Data Collection Survey: the incidence and geographical distribution of acute myeloid leukemia. *Leukemia* **3**: 875–879

Öst Å, Lindström P, Christensson B, Gyllenhammar H and Engstedt L (1984) Acute leukaemia in a defined geographic area – incidence, clinical history and prognosis. *Scand J Haematol* **33**: 160–170

Proctor SJ, Taylor PRA, Stark A, Carey PJ, Bown N, Hamilton PJ and Reid MM (1995) Evaluation of the impact of allogenic transplant in first remission on an unselected population of patients with acute myeloid leukaemia aged 15–55 years. *Leukemia* **9**: 1246–1251

Rowe JM and Liesveld JL (1996) Treatment and prognostic factors in acute myeloid leukaemia. *Baillières Clin Haematol* **9**: 87–105

Statistics Sweden (1987–1992) *DEMOPAK*

Taylor PRA, Reid MM, Stark AN, Bown N, Hamilton PJ and Proctor SJ (1995) De novo acute myeloid leukaemia in patients over 55-years-old: a population-based study of incidence, treatment and outcome. *Leukemia* **9**: 231–237

The Toronto Leukemia Study Group (1986) Results of chemotherapy for unselected patients with acute myeloblastic leukaemia: effect of exclusions on interpretation of results. *Lancet* **i**: 786–788

Wahlin A, Hörnsten P and Jonsson H (1991) Remission rate and survival in acute myeloid leukemia: impact of selection and chemotherapy. *Eur J Haematol* **46**: 240–247

 *© 2000 Cancer Research Campaign*
